# The midgut of *Aedes albopictus* females expresses active trypsin-like serine peptidases

**DOI:** 10.1186/1756-3305-7-253

**Published:** 2014-05-30

**Authors:** Leonardo Saboia-Vahia, Patricia Cuervo, Andre Borges-Veloso, Nathália Pinho de Souza, Constança Britto, Geovane Dias-Lopes, Jose Batista De Jesus

**Affiliations:** 1 Laboratório de Biologia Molecular e Doenças Endêmicas, Instituto Oswaldo Cruz, FIOCRUZ, Rio de Janeiro, Brazil; 2 Laboratório de Pesquisa em Leishmaniose, Instituto Oswaldo Cruz, FIOCRUZ, Rio de Janeiro, Brazil; 3 Universidade Federal de São João del Rei, São João del Rei, MG, Brazil

**Keywords:** *Aedes albopictus*, Culicidae, Midgut, Zymography, Proteomics, Two-dimensional electrophoresis, Mass spectrometry

## Abstract

**Background:**

*Aedes albopictus* is widely distributed across tropical and sub-tropical regions and is associated with the transmission of several arboviruses. Although this species is increasingly relevant to public health due its ability to successfully colonize both urban and rural habitats, favoring the dispersion of viral infections, little is known about its biochemical traits, with all assumptions made based on studies of *A. aegypti*. In previous studies we characterized the peptidase profile of pre-imaginal stages of *A. albopictus* and we reported the first proteomic analysis of the midgut from sugar-fed females of this insect species.

**Methods:**

In the present work, we further analyzed the peptidase expression in the midgut of sugar-fed females using 1DE-substrate gel zymography, two-dimensional electrophoresis (2DE), mass spectrometry (MS), and protein identification based on similarity.

**Results:**

The combination of zymography, in solution assays using fluorescent substrates and 2DE-MS/MS allowed us to identify the active serine peptidase “fingerprint” in the midgut of *A. albopictus* females. Zymographic analysis revealed a proteolytic profile composed of at least 13 bands ranging from ~25 to 250 kDa, which were identified as trypsin-like serine peptidases by using specific inhibitors of this class of enzymes. Concomitant use of the fluorogenic substrate Z-Phe-Arg-AMC and trypsin-like serine protease inhibitors corroborated the zymographic findings. Our proteomic approach allowed the identification of two different trypsin-like serine peptidases and one chymotrypsin in protein spots of the alkaline region in 2DE map of the *A. albopictus* female midgut. Identification of these protein coding genes was achieved by similarity to the *A. aegypti* genome sequences using Mascot and OMSSA search engines.

**Conclusion:**

These results allowed us to detect, identify and characterize the expression of active trypsin-like serine peptidases in the midgut of sugar-fed *A. albopictus* females. In addition, proteomic analysis allowed us to confidently assign the expression of two trypsin genes and one chymotrypsin gene to the midgut of this mosquito. These results contribute to the gene annotation in this species of unknown genome and represent a small but important step toward the protein-level functional and localization assignment of trypsin-like serine peptidase genes in the *Aedes* genus.

## Background

*Aedes* (Stegomyia) *albopictus* (Skuse) has a wide geographic distribution, covering all tropical and subtropical regions of the world, and is a vector for the viruses responsible for yellow fever and dengue [1]. The World Health Organization estimates that more than 50–100 million cases of these two diseases can occur per year throughout the world [2-4]. In Brazil, *A. albopictus* has been reported in 21 states and 1,502 municipalities [5]. In recent years, the relevance of this species to public health has increased because it is able to successfully colonize both urban and rural habitats, favoring the dispersion and interchange of the virus from one area to another, and thereby enabling the emergence of new areas of disease in small and large cities [6,7].

The hydrolysis of proteins to amino acid residues by proteolytic enzymes is an important step in food digestion, protein turnover and proteostasis in eukaryotes [8,9]. Proteolytic enzymes are divided into endopeptidases and exopeptidases. Endopeptidases are relatively small molecules (~25-30 kDa) that can pass through peritrophic membrane pores and endoperitrophic spaces in insects, where they cleave large protein complexes. Exopeptidases are large enzymes (>100 kDa) that are usually linked to the plasma membrane of the midgut epithelium and hydrolyze the ends of small proteins and peptides (N-terminus or C-terminus) [10]. Among endopeptidases, trypsin-like and chymotrypsin-like serine peptidases are the most important enzymes for most insects, except for some species of coleoptera and hemiptera [11-16].

Serine peptidases are divided into families and subfamilies. The subfamily S1 consists of trypsins, chymotrypsins and elastases, and some serine collagenases were also recently included. The catalytic triad of serine peptidases is typically characterized by serine, histidine and aspartic acid residues [17,18]. This triad hydrolyzes peptide bonds at the carboxylic ends of basic amino acids, with a 2-10-fold preference for Arg over Lys [19-21]. The *A. aegypti* genome contains 369 genes coding for serine peptidases, among which 66 are putative trypsins [22], but only 5 (three trypsins and two chymotrypsins) are well characterized in the midgut of females of this insect [13-16,23]. The expansion of trypsin-like serine peptidase genes in mosquitoes has been shown to coincide with the development of the hematophagous trait [24]. Trypsin-like serine peptidases in these insects play pivotal roles in oogenesis, immunity, metamorphosis, modulation of embryonic development and nutrition [25,26]. These enzymes are mostly located in the insect midgut so that they can provide energy and essential amino acids for development [20,27]. Furthermore, secretion of trypsin-like serine peptidases into the lumen of the midgut is involved in defense against pathogens [28,29]. However, in the insect vector *Anopheles gambiae*, trypsin-like enzymes are exploited by pathogens such as *Plasmodium* sp. to activate their own peptidases, thus allowing the parasite to cross the peritrophic membrane and continue its developmental life cycle [30].

The plasticity exhibited by trypsin-like serine peptidases enables these enzymes to modulate various biological processes in insect vectors. Because of this characteristic, serine peptidases have been proposed as potential targets for insect control approaches. The biochemical characterization of these enzymes may thus support the development of new control strategies, enabling their appropriate use as targets and suggesting ways to interfere with the production of these enzymes or with the metabolic pathways in which they participate [31,32].

In previous studies, we characterized the peptidase profile of the pre-imaginal stages of *A. albopictus*[33] and reported the first proteomic analysis of the midgut of sugar-fed females of this insect species [33]. In the present work, we further analyze the peptidase expression in the midgut of sugar-fed females using two-dimensional electrophoresis (2DE), mass spectrometry, 1DE-substrate gel, and data mining. This multi-methodological approach allowed us to identify the active serine peptidase “fingerprint” in the midgut of *A. albopictus* females.

## Methods

### Chemicals

To prepare the phenylmethylsulfonylfluoride (PMSF) stock solution, 250 mM of the reagent was diluted in isopropanol. Nα-tosyl-L-lysine chloromethyl ketone hydrochloride (TLCK) and N-p-tosyl-L-phenylalanine chloromethyl ketone (TPCK) were dissolved in methanol, both at 100 mM. Stock solutions of 1,10-phenanthroline (200 mM) and pepstatin A (1 mg/ml) were prepared in ethanol, and *trans*-epoxysuccinyl L-leucylamido-(4-guanidino) butane (E-64, 10 mM) was prepared in water. Stock and working solutions were maintained at -20°C. All chemicals were purchased from Sigma Chem. Co. (USA), unless otherwise specified.

### Insect rearing and gut dissection

*Aedes albopictus* specimens reared in a closed colony (Laboratório de Transmissores de Hematozoários, Instituto Oswaldo Cruz, FIOCRUZ, Rio de Janeiro) were kindly provided by Dr. Nildimar A. Honorio. Mosquitoes were maintained on a 10% sucrose diet at 25 ± 1°C, with a relative humidity of 60 ± 10% and a light:dark photoperiod of 14:10 h. For each experiment, 50 female adults (2–5 days old) were cold-anesthetized on ice and decapitated. Midgut dissection was performed as previously described [33].

### Zymography and peptidase inhibition assays

For proteolytic assessment, midguts were washed twice with PBS pH 7.2 and lysed as previously described [33]. Briefly, midguts were lysed with a cell disruption motor drive and pestle in a tube containing 10% glycerol, 0.6% Triton X-100, 100 mM Tris–HCl pH 6.8 and 150 mM NaCl [34]. The protein concentration of the resulting extracts was determined using the Pierce 660 nm Protein assay (Thermo Scientific). For protein separation, 30 μg of protein were loaded in 12% polyacrylamide gels copolymerized with 0.1% porcine gelatin as the substrate. Electrophoreses were performed at 4°C with a constant voltage of 110 V. Peptidase activity was detected as previously reported [33] with few modifications. The gels were incubated at 37°C for 2, 4, 6, 12 or 24 h in reaction buffer containing 100 mM sodium acetate (at pH 3.5 or 5.5) or 100 mM Tris–HCl (pH 7.5 or 10.0). Substrate degradation was visualized as clear bands after staining the gels with 0.2% Coomassie blue R-250 in methanol/acetic acid (40:10) and destaining in 10% acetic acid. The relative molecular masses of the activity bands were estimated by comparison with the mobility of a commercial molecular mass standard (PageRuler™ Protein Ladder, Fermentas). To determine the classes of peptidases detected by zymography, peptidase inhibition assays were conducted. Midgut homogenates were pre-incubated (before electrophoresis) for 30 min at 4°C with one of the following peptidase inhibitors: 20 μM E-64, 5 mM PMSF, 100 μM TLCK, 100 μM TPCK, 10 μM pepstatin-A or 10 mM 1,10-phenanthroline. After electrophoresis, inhibitors were added to the reaction buffer at the same concentration, and the peptidases were resolved as described above. The results were derived from three independent experiments carried out in triplicate.

### In-solution enzymatic assays

The effects of pH and peptidase inhibitors on the proteolytic activities of midgut homogenates were also evaluated by in-solution assays using the fluorogenic substrate Z-Phe-Arg-AMC. For both assays, 100 μM of substrate was used. The reactions were initiated by diluting 10 μg of protein from the midgut in 100 mM sodium acetate (at pH 3.5 or 5.5) or 100 mM Tris–HCl (pH 7.5 or 10.0) for pH evaluation or 100 mM Tris–HCl pH 7.5 with or without 100 μM TLCK, 100 μM TPCK, 20 μM E-64 or 5 mM PMSF. The fluorescence intensity was measured by spectrophotofluorometry every 5 min for a 60 min period (SpectraMax Gemini XPS, Molecular Devices, CA) using excitation and emission wavelengths of 380 and 460 nm, respectively. As blank, the substrate (100 μM) was diluted in the reaction buffer [100 mM sodium acetate (at pH 3.5 or 5.5) or 100 mM Tris–HCl (pH 7.5 or 10.0)]. The value of the blank was automatically discounted by the fluorometer software (SoftMax®Pro, Molecular Devices, CA) when the data were acquired. All assays were performed at 37°C. The results were derived from three independent experiments performed in triplicate.

### 2DE electrophoresis and protein identification

Protein extraction, separation and identification were performed as previously described [33]. Briefly, 50 pooled midguts were mechanically disrupted with a pestle and a motor drive in a tube containing lysis buffer (9 M urea, 4% CHAPS, 65 mM dithiothreitol, DTT, and 1% ampholytes, pH 3–10, with 5 mM PMSF and a protease inhibitor cocktail). Proteins were precipitated and resuspended in 9 M urea, 4% CHAPS, 65 mM DTT and 1% ampholytes, pH 3–10 NL. The protein concentration was determined using the 2D Quant Kit (GE Healthcare), and 100 μg were subjected to isoelectric focusing over a nonlinear pH gradient of 3–10 on a 7 cm strip (GE Healthcare) on an Ettan IPGphor 3 instrument (GE Healthcare). The focusing parameters were set as previously described [33]. After reduction and alkylation, proteins were separated vertically across 12% SDS-PAGE gels using standard Tris/glycine/SDS buffer. Gels were stained with colloidal Coomassie Brilliant Blue G-250, documented using a GS-800™ calibrated imaging densitometer (Bio-Rad) and analyzed using PDQuest™ software (Bio-Rad). Experimental p*I* and *M*r were calibrated using a select set of reliable identification landmarks distributed throughout the entire gel.

Protein digestion, peptide extraction and analysis by mass spectrometry were performed as previously described [33]. Raw MS files were converted to MGF format using Mass Matrix MS Data File converter V. 3.9 http://www.massmatrix.net/mm-cgi/downloads.py. To maximize search sensitivity and peptidase identification, the data were searched using OMSSA [35] within the Proteomatic platform 1.2.1 [36]http://www.proteomatic.org/download.html and the Mascot MS/MS ion search engine (http://www.matrixscience.com/search_form_select.html, Matrix Science, Oxford, UK, free online version). As the genome sequences of *A. albopictus* are not available, mass spectra were searched in OMSSA against an *A. aegypti* database downloaded (May 2013) from UniRef100 [37]http://www.uniprot.org/, and in MASCOT against the non-redundant database of the National Center for Biotechnology (NCBI). Searches were performed with one missed cleavage, with carbamidomethylation of cysteine residues as a fixed modification, methionine oxidation as a variable modification and mass tolerances of 2.0 and 0.8 Da in OMSSA and 10 ppm and 0.4 Da in MASCOT for precursor and fragment ions, respectively. The Vectorbase database (http://www.vectorbase.org) was used to search for sequence information about the identified peptidases.

## Results and discussion

### The *A. albopictus* female midgut exhibits a complex profile of active peptidases

**Figure 1:**
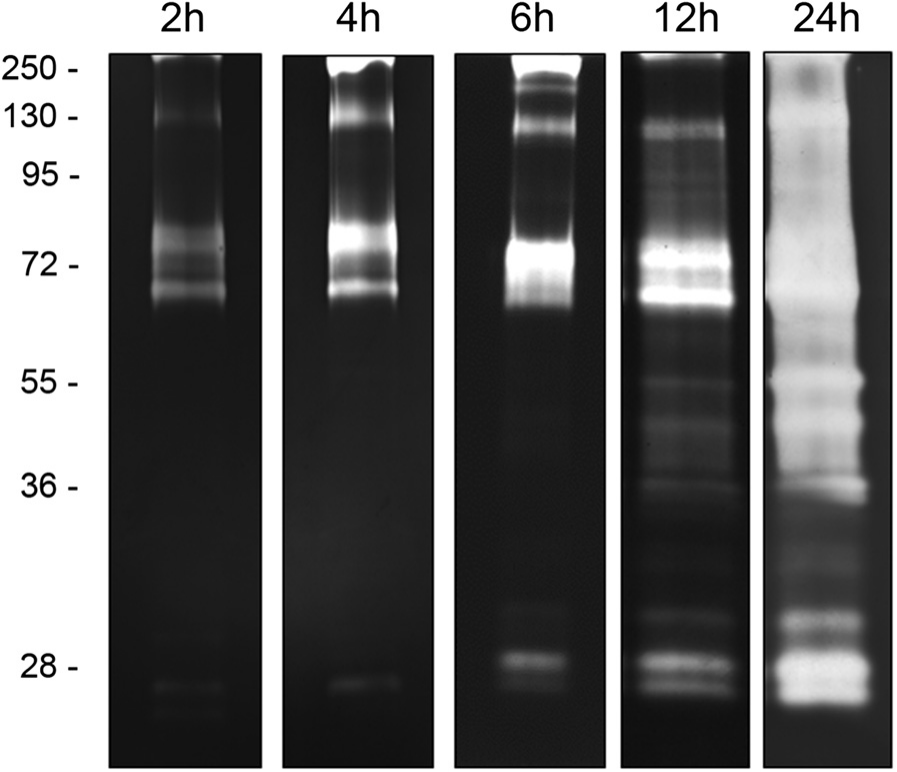
**Time course of the proteolytic activities exhibited by the *A. albopictus *midgut.** Proteolytic activities were evaluated after 2, 4, 6 12 and 24 h of incubation in 0.1 M Tris–HCl buffer (pH 7.5). The numbers on the left indicate the apparent molecular masses of the peptidases (kDa).

To evaluate the influence of time on the peptidase activities from the midguts of sugar-fed females, we evaluated the proteolytic profiles after 2, 4, 6 12 and 24 h of incubation at 37°C in pH 7.5 using 1D zymography (Figure 1). The proteolytic profile is composed of at least 13 bands displaying progressive increases in intensity from 2 to 24 h of incubation. However, the proteolytic bands overlapped at 24 h and for this reason, 12 h was chosen for subsequent analyses. The band size range from ~25 to 250 kDa. The proteolytic activities of this mosquito have received little study, focusing mainly on larval activities and on comparisons with *A. aegypti*[38]. Comparisons between the previously reported peptidase profiles from the larval and pupal stages of *A. albopictus*[33] with the profile from the adult midgut obtained here reveals a similar banding pattern of enzymatic activity, suggesting that the peptidases expressed as part of the adult proteolytic machinery are already expressed in the pre-imaginal stages, where they mainly seem to play roles in digestion. Given that this work studied sugar-fed females, the proteolytic profile detected here is likely constitutively expressed by the mosquito throughout its life cycle. The enzymatic activity profile of the adult midgut was more complex after 12 h of reaction compared with the profile previously described for larvae [33] detected after 2 h of reaction, suggesting that these enzymes are expressed at lower levels in the adult stage of this insect. In fact, a reduction in the expression of active peptidases involved in digestion after the last larval ecdysis has been reported for *A. aegypti* and *A. albopictus* using in-solution assays and total protein extracts [39,40]. In sugar-fed females of *A. aegypti*, the expression of trypsin-like serine peptidases at the transcriptional level (“early trypsin”) has been associated with the activation of other digestive enzymes or other trypsin isoforms that will only be expressed after the ingestion of a blood meal [23,27,41]. In addition, although transcriptomic analysis of *A. aegypti* revealed that adults exclusively express 15 trypsin genes while larvae express 12, both stages share the expression of 39 trypsin coding genes, and the genes expressed by larvae should be more active than those of adults because larvae are constantly feeding [22]. Given the taxonomic proximity between *A. albopictus* and *A. aegypti*, trypsin expression in adults of *A. albopictus* is likely similar to the expression pattern in larvae.

### Influence of the pH on the proteolytic profile of the *A. albopictus* midgut

**Figure 2:**
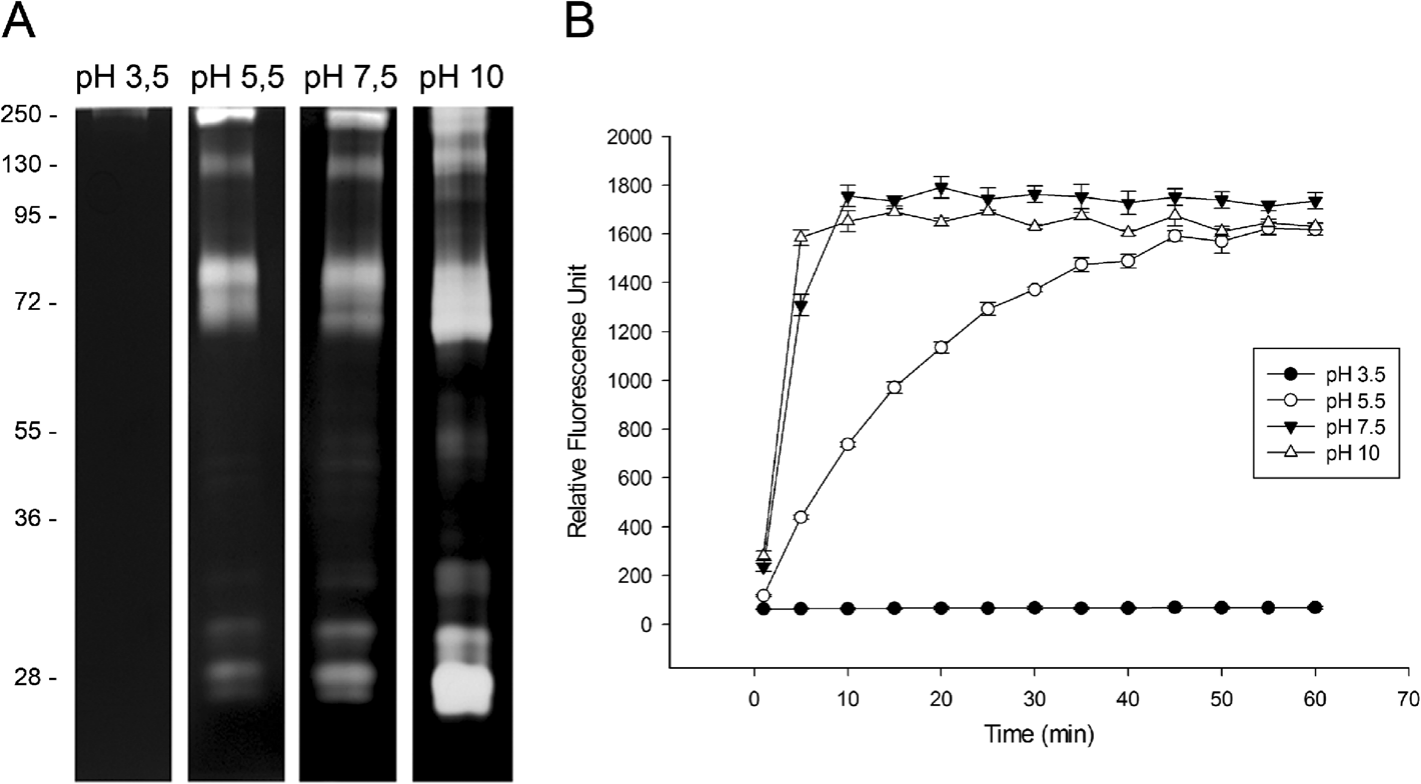
**Influence of pH and temperature on the proteolytic profiles of the *A. albopictus *midgut.** The effect of pH on the proteolytic activities was evaluated by the incubation of protein extracts in 0.1 M sodium acetate buffer pH 3.5 or 5.5 or 0.1 M Tris–HCl buffer pH 7.5 or 10.0. **(A)** Zymographic gels incubated at 37°C for 12 h. The numbers on the left indicate the apparent molecular masses of the peptidases (kDa). **(B)** In-solution assays performed using the fluorogenic substrate Z-Phe-Arg-AMC.

We observed that the proteolytic activities of the midgut are modulated by the pH of the reaction buffer. While peptidase activities were detected as low as pH 5.5, the intensity of these activities was increased at alkaline pH between 7.5 and 10.0. Little activity was detected at pH 3.5 (Figure 2A). Although the activities seem to be optimal at pH 10.0, several bands overlap, preventing accurate analysis. For this reason, all subsequent assays were conducted at pH 7.5. The effect of pH on peptidase activity was corroborated by in-solution assays using fluorogenic substrates (Figure 2B). Similar to data obtained previously for larval instars and pupae [33], peptidases from the *A. albopictus* adult midgut exhibited optimal activities at alkaline pH. This characteristic has also been observed for other Diptera such as *Culex quinquefasciatus*[5] and *L. longipalpis*[42] using both in-gel and in-solution assays. According to previous reports, the activities of enzymes involved in digestion processes are optimal at alkaline pH [37-39]. Therefore, the data obtained here suggest that the proteolytic activities observed in the midgut are mainly involved in nutrient processing.

### Proteolytic activities in the *A. albopictus* female midgut mainly arise from trypsin-like serine peptidases

**Figure 3:**
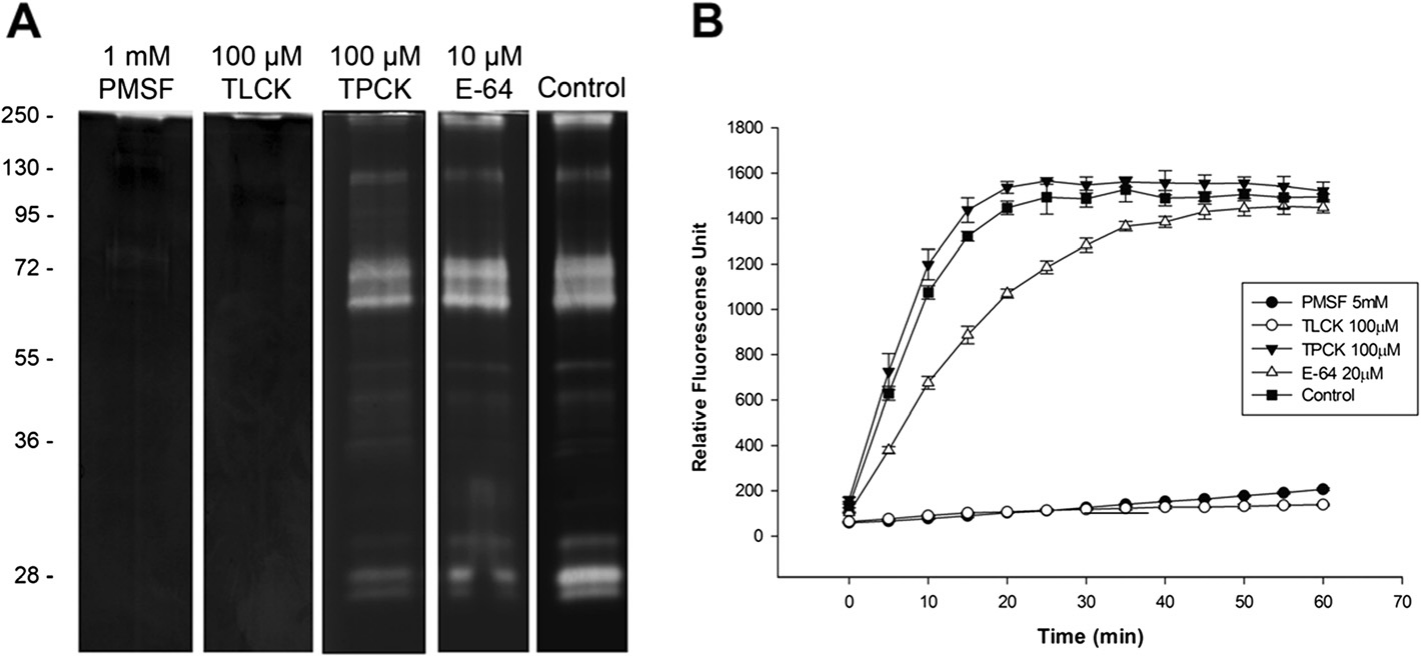
**Effects of peptidase inhibitors on the proteolytic profiles of the *A. albopictus *midgut and quantitative in-solution assays for the proteolytic activities. (A)** Samples were pre-incubated for 30 min in the presence of 1 mM PMSF, 100 μM TLCK, 100 μM TPCK and 10 μM E-64. The proteolytic activities were detected after incubating the gels for 12 h at 37°C in Tris–HCl buffer, pH 7.5. The control samples were processed in the same way but in the absence of inhibitors. The numbers on the left indicate the apparent molecular masses of the peptidases (kDa). **(B)** The in-solution assays were performed using the fluorogenic substrate Z-Phe-Arg-AMC in the absence (control) or presence of 5 mM PMSF, 100 μM TLCK, 100 μM TPCK and 20 μM E-64 in 100 mM Tris–HCl buffer, pH 7.5.

Inhibition assays were conducted using specific peptidase inhibitors to ascertain the classes of peptidase activities detected in the midgut of *A. albopictus* females. The proteolytic activities were inhibited by 1 mM PMSF and 100 μM TLCK (Figure 3A). The zymographic profile was not affected by 100 μM TPCK, 10 μM E-64 (Figure 3A), 10 μM pepstatin A or 10 mM 1,10-phenanthroline (data not shown). As PMSF and TLCK are inhibitors of serine peptidases and trypsin-like serine peptidases, respectively, these results indicate that the main peptidases detected by zymography in the female midgut are trypsin-like serine peptidases. Protein extracts from the female midgut were also reacted with the fluorogenic substrate Z-Phe-Arg-AMC in the presence or absence of 5 mM PMSF, 100 μM TLCK, 100 μM TPCK or 20 μM E-64. These activities were again strongly inhibited by PMSF and TLCK, but not by TPCK, (Figure 3B) corroborating the in-gel results. The occurrence of trypsin-like serine peptidases has been described in the larvae of *A. aegypti* and *A. albopictus* using both in-solution assays [38,39] and zymographic analysis [33,43]. The main peptidases detected in other Diptera species also belong to the serine peptidase class [42,44,45].

### Trypsin-like serine peptidases and chymotrypsin were identified in the 2DE map of the *A. albopictus* female midgut

**Figure 4:**
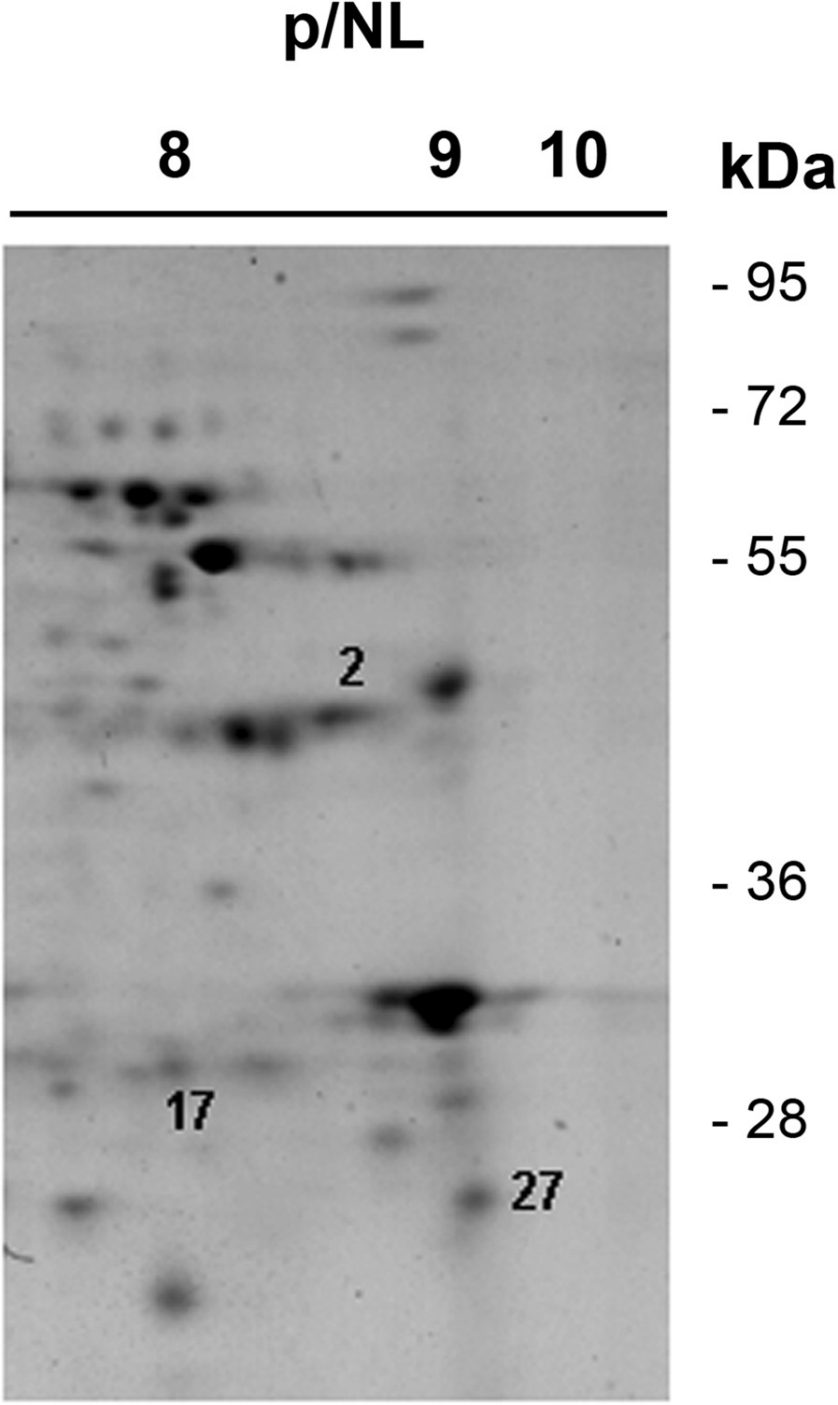
**Alkaline region of a 2DE map of soluble proteins from the midgut of *Ae. albopictus *females.** Proteins were separated in the first dimension across a non-linear pH range of 3–10 NL, and in the second dimension by 12% SDS-PAGE [33]. Protein spot numbers correspond to: [2] trypsin-alpha, [17] trypsin, and [27] chymotrypsin. Details of the identification are provided in Table 1. The numbers on the right side indicate the molecular mass standards in kDa.

**Figure 5:**
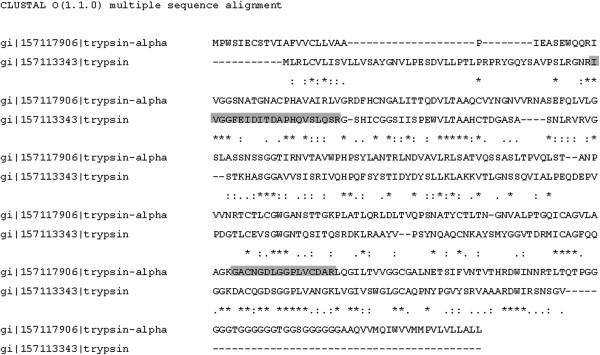
**Sequence alignment of trypsin-alpha and trypsin genes detected in the midgut of *A. albopictus*.** Identical residues are marked with an asterisk; residues with strongly similar properties are marked with a colon; residues with slightly similar properties are indicated with a period. Peptides identified in each protein by mass spectrometry are highlighted in gray.

With the aim of identifying the peptidases detected in the zymographic assays, several protein spots resolved in the alkaline region of 2D gel maps from the *A. albopictus* female midgut previously obtained and reported by our group [33] were excised and analyzed by MS. Two protein spots corresponding to trypsin alpha (AAEL008079) and trypsin (AAEL006425), respectively, and one spot corresponding to chymotrypsin (AAEL009680) were identified by their similarity to the *A. aegypti* sequences through the two search engines used for protein identification (Figure 4, Table 1). When the protein spots identified in the 2DE gel were compared to the proteolytic profile by 1D zymography, we observed that the molecular masses of peptidases identified by 2DE correspond to the apparent molecular masses of some of the proteolytic bands. The sequence alignment of both trypsin genes demonstrates very little similarity among the sequences. In addition, the peptide sequences identified by MS/MS for each gene are also different (Figure 5). Interestingly, while a chymotrypsin gene was identified by MS/MS in the midgut 2DE map, we did not observe this peptidase activity in the zymographic assays. This result may indicate that (i) this enzyme is expressed in the midgut of sugar-fed females but is not active under these conditions, *i.e.*, it may be active only under blood feeding condition; or (ii) that, despite being expressed, the chymotrypsin is not active in the adult stage of the insect, *i.e.*, this enzyme could be active only in other stages of the insect life cycle.

**Table 1:** ***Aedes albopictus *** **midgut peptidases automatically identified using the Mascot software**

**Spot code**	**Protein name**	**NCBI Accesion No.**	**VectorBase DB No.**	**Theor/Exp MW**	**Theor/Exp p/**	**Matching pep./ Pep. identified by MS/MS**	**Peptide sequence**	**Error ± ppm**	**Protein score**	**Ion score**
27	Chymotrypsin, putative [Aedes aegypti]	gi|157123854	AAEL009680	26.5/25.8	9.0/9.3	1(1)	R.SNELQTLYQK.T	3	64	64
2	Trypsin-alpha, putative [Aedes aegypti]	gi|157117906	AAEL008079	32.1/40.8	8.0/8.8	1(1)	K.GACNGDLGGPLVCDAR.L	3	78	78
17	Trypsin [Aedes aegypti]	gi|157113343	AAEL006425	29.6/29.6	8.6/8.2	1(1)	R.IVGGFEIDITDAPHQVSLQSR.G	4	106	106

Spot codes correspond to spot numbers in Figure 4. MW: molecular weight. p*I*: isoelectric point. ppm: parts per million.

Although the *A. aegypti* genome has been reported to code for 380 trypsin-like serine peptidases, constituting one of the largest gene families in mosquitoes [22], the interrogation of the Vectorbase database using the words “trypsin” or “chymotrypsin” reveals only 80 coding genes for trypsin and 5 coding genes for chymotrypsin in the *A. aegypti* genome, among which are the genes identified here. As these peptidases are members of large gene families, it is difficult to ascertain which protein is expressed and active in a specific life cycle stage, in a specific tissue, under a specific condition. Therefore, we cannot rule out the possibility that other proteases, such as chymotrypsin, could be expressed in their active form in the midgut of blood feeding females. Our study shows the potential value of proteomic approaches combined with zymographic analysis for the identification and localization assignment of specific gene products.

## Conclusion

The results obtained in this work allowed us to detect, identify and characterize the expression of active trypsin-like serine peptidases in the midgut of sugar-fed *A. albopictus* females. In addition, proteomic analysis allowed us to confidently assign the expression of two trypsin genes and one chymotrypsin gene to the midgut of this mosquito. These results contribute to the gene annotation in this species of an unknown genome and represent a small but important step toward the protein-level functional assignment of trypsin-like serine peptidase genes in the *Aedes* genus. As peptidases exert crucial roles during host-pathogen interactions and the midgut is the main setting for these interactions in blood feeding vector mosquitoes, the mapping and identification of the constitutively expressed peptidase profile in this tissue may allow for the comparison of the regulation of such enzymes in infected insects and/or mosquitoes fed on blood. Such approaches may produce valuable information on the roles of peptidases during host-pathogen interactions.

## Competing interests

The authors declare that they have no competing interests.

## Authors’ contributions

JBJ, LSV and PC designed the study. LSV, ABV, NPS and GDL performed the experimental work. LSV, PC and JBJ analyzed the data and prepared the manuscript with the critical input of CB. All authors read and approved the final manuscript.
